# Combined Impact of Cardiorespiratory Fitness and Visceral Adiposity on Metabolic Syndrome in Overweight and Obese Adults in Korea

**DOI:** 10.1371/journal.pone.0085742

**Published:** 2014-01-15

**Authors:** Sue Kim, Ji-Young Kim, Duk-Chul Lee, Hye-Sun Lee, Ji-Won Lee, Justin Y. Jeon

**Affiliations:** 1 Department of Family Medicine, Severance Hospital, Yonsei University College of Medicine, Seoul, Korea; 2 Department of Sport and Leisure Studies, Yonsei University, Seoul, Korea; 3 Biostatistics Collaboration Units, Department of Research Affairs, Yonsei University College of Medicine, Seoul, Korea; University of Sao Paulo, Brazil

## Abstract

**Background:**

Obesity, especially visceral obesity, is known to be an important correlate for cardiovascular disease and increased mortality. On the other hand, high cardiorespiratory fitness is suggested to be an effective contributor for reducing this risk. This study was conducted to determine the combined impact of cardiorespiratory fitness and visceral adiposity, otherwise known as fitness and fatness, on metabolic syndrome in overweight and obese adults.

**Methods:**

A total of 232 overweight and obese individuals were grouped into four subtypes according to their fitness level. This was measured by recovery heart rate from a step test in addition to visceral adiposity defined as the visceral adipose tissue area to subcutaneous adipose tissue area ratio (VAT/SAT ratio). Associations of fitness and visceral fatness were analyzed in comparison with the prevalence of metabolic syndrome.

**Results:**

The high visceral fat and low fitness group had the highest prevalence of metabolic syndrome [Odds Ratio (OR) 5.02; 95% Confidence Interval (CI) 1.85–13.61] compared with the reference group, which was the low visceral adiposity and high fitness group, after adjustments for confounding factors. Viscerally lean but unfit subjects were associated with a higher prevalence of metabolic syndrome than more viscerally obese but fit subjects (OR 3.42; 95% CI 1.27–9.19, and OR 2.70; 95% CI 1.01–7.25, respectively).

**Conclusions:**

Our study shows that visceral obesity and fitness levels are cumulatively associated with a higher prevalence of metabolic syndrome in healthy overweight and obese adults. This suggests that cardiorespiratory fitness is a significant modifier in the relation of visceral adiposity to adverse metabolic outcomes in overweight and obese individuals.

## Introduction

Obesity is a major cause of metabolic dysregulation and leads to increased morbidity and mortality; therefore, it is a significant health concern worldwide [1], [2]. Among obese characteristics, the accumulation of abdominal visceral adiposity has been shown to be strongly associated with a cluster of metabolic abnormalities and contributes to insulin resistance and metabolic syndrome [3], [4]. Furthermore, visceral adiposity is an independent risk factor for cardiovascular diseases, such as hypertension and diabetes, irrespective of total body fat [5], [6].

On the other hand, increased energy expenditure through physical activity prevents individuals from developing obesity and its related risks of metabolic abnormalities [7], [8]. Cardiorespiratory fitness, or aerobic fitness, refers to the ability of circulatory and respiratory function to supply oxygen to muscles and other organs during sustained physical activity without tiring out [9]. Increased cardiorespiratory fitness is known to reduce the risk of cardiometabolic diseases and can be estimated with recovery heart rate after exercise quantified as the indicator for physical fitness level [10], [11]. Recent studies demonstrate that a greater level of measured recovery heart rate is associated with an increased risk for metabolic syndrome as well as cardiovascular diseases and all-cause mortality [12]–[14].

The effects of obesity and fitness as unfavorable and favorable contributors to the risk of developing cardiometabolic diseases have become of clinical interest. Indeed, some studies exploring the combination of fitness and fatness on metabolic disturbance and mortality have shown that fitness was a powerful modifier of this association [15], [16]. Moreover, overweight but physically active individuals were shown to have an equivalent or even lower risk of death than lean but inactive individuals [17], [18]. However, although central visceral adiposity is a critical determinant of metabolic profiles in obesity, little is known about visceral adiposity, cardiorespiratory fitness, and their combined impact on metabolic profiles in overweight and obese subjects.

We hypothesized that overweight and obese adults with high visceral adiposity and low fitness level would have high prevalence of metabolic syndrome, and furthermore, the combination of the two factors would be related to the magnitude of the association on the prevalence of metabolic syndrome. Therefore, this study was conducted to investigate the combined impact of visceral obesity and cardiorespiratory fitness on metabolic syndrome in overweight and obese adults as determined by abdominal computed tomography (CT) scan measurement and heart rate recovery after exercise.

## Materials and Methods

### Ethics Statement

All subjects participated in the study voluntarily, and written informed consent was obtained from each participant. The study complied with the Declaration of Helsinki and was approved by the Institutional Review Board of Severance Hospital.

### Study participants

A total of 232 men and women participated in this substudy of the Korean Physical Activity and Obesity (K-POP) study, which is an ongoing study designed to examine physical fitness and metabolic markers of overweight and obese adults in Seoul, Korea. The participants were recruited from the visitors to the obesity clinic in the department of family medicine at Severance Hospital from January 2010 to March 2013.

Being overweight was defined as having a body mass index (BMI) ≥ 23 kg/m^2^, and obesity was defined as having a BMI ≥ 25 kg/m^2^, following Asian-pacific population specific BMI criteria by World Health Organization expert consultation [19]. Only healthy subjects aged 18 to 70 years without underlying medical conditions were included in this study, such that those with histories of hypertension, diabetes, dyslipidemia, chronic liver disease or other cardiovascular diseases were excluded. Those with medications that can affect cardiometabolic function, including anti-hypertensives, hypoglycemic agents, or anti-obesity drugs, were also excluded from the study. Participants who were not able to fulfill the cardiorespiratory fitness evaluation due to their physical or psychological conditions were likewise excluded.

### Clinical and anthropometric evaluation

Data on past and current medical conditions and medications were collected from medical records. BMI was calculated as weight divided by height squared. Body weight was measured to the nearest 0.1 kg with an electronic scale, and height was measured to the nearest 0.1 cm with a stadiometer. Waist circumference was measured midway between the lowest rib and the iliac crest in the standing position. Blood pressure was measured two times by mercury sphygmomanometer after at least 10-min seated rest, and the average of the two measurements was recorded. Mean blood pressure was calculated based on this measurement. Lifestyle factors such as smoking status, alcohol consumption, and physical activity status were provided by participants through questionnaires. Smoking status was considered “yes” if the subjects reported themselves as a current smoker. Alcohol consumption was defined as a positive factor if alcohol consumption was 72 g or more per week. Physical activity status was analyzed from a participant’s overall energy expenditure calculated in metabolic equivalents hour (MET-h) per week from information acquired through the Korean version of the International Physical Activity Questionnaire (IPAQ) [20].

Abdominal adipose tissue area was quantified by CT (Tomoscan 350; Philips, Mahwah, NJ, USA). Specifically, a 10-mm CT slice scan was acquired at the L4–L5 level with subjects in the supine position in order to measure the total abdominal tissue (TAT) and visceral adipose tissue (VAT) area. VAT was quantified by delineating the intra-abdominal cavity at the internal aspect of the abdominal and oblique muscle walls surrounding the cavity and the posterior aspect of the vertebral body. The subcutaneous adipose tissue (SAT) area was calculated by subtracting the VAT area from the TAT area. The coefficients of variation for inter- and intra-observer reproducibility for VAT were 1.4% and 0.5%, respectively.

### Biochemical analyses

Biochemical analyses were performed on blood samples collected after overnight fasting (>12 hrs). Serum levels of fasting glucose, total cholesterol, triglycerides, high-density lipoprotein cholesterol, low-density lipoprotein cholesterol, and high-sensitivity C-reactive protein were measured with the Hitachi 7600 Automatic analyzer (High-Technologies Corporation, Hitachi, Tokyo, Japan). Fasting insulin was measured by electrochemiluminescence immunoassay using an Elecsys 2010 (Roche, Indianapolis, IN, USA), and insulin resistance was estimated using the homeostasis model assessment of insulin resistance (HOMA-IR) index [Insulin (mUl/L) × fasting glucose (mg/dl)/405] [21].

Metabolic syndrome was defined using the criteria proposed by the American Heart Association and the National Heart, Lung, and Blood Institute, with waist circumference criteria modification based on the following World Health Organization-Asian Pacific region criteria for abdominal obesity [19], [22]: (1) a waist circumference ≥90 cm for men and ≥85 cm for women; (2) triglycerides ≥150 mg/dl; (3) serum HDL cholesterol <40 mg/dl for men and <50 mg/dl for women; (4) systolic blood pressure ≥130 mmHg, diastolic blood pressure ≥85 mmHg, or use of anti-hypertensive medication); and (5) fasting glucose ≥100 mg/dl, or insulin or hypoglycemic medication use.

### Cardiorespiratory fitness

Cardiorespiratory fitness was evaluated using Tecumseh, a standardized 3-minute step test. Participants performed 24 steps per minute based on the protocol for the Tecumseh step test, maintaining a constant stepping rate on a 20.3 cm-high step for 3 minutes [10], [23]. The participants were aided by an assistant’s demonstration and a metronome cadence for proper stepping technique and constant step maintenance. Heart rates were measured and recorded by a heart rate monitor (Polar-FS3C, USA) attached on the anterior chest wall of the participant. Heart rates were recorded in a seating position 1 minute prior to exercise after at least 5 minutes of resting and 1 minute after the completion of the 3-minute step exercise (1 minute recovery heart rate). The expectation was that participants with greater cardiorespiratory fitness would have lower heart rates at 1 minute post-exercise than those with worse cardiorespiratory fitness [11].

### Statistical analyses

To examine the joint impact of visceral fat and fitness level on the prevalence of metabolic syndrome, participants were divided into the following four groups according to the combination of their visceral obesity and fitness level: (1) low visceral fat and high level of fitness, (2) low visceral fat and low level of fitness, (3) high visceral adiposity and high level of fitness, and (4) high visceral adiposity and low level of fitness. Low visceral obesity was defined as having a VAT/SAT ratio measured by CT scan of less than 0.4, and high visceral obesity was defined as having a VAT/SAT ratio ≥ 0.4 [24]. Fitness level was divided into high or low according to the median value (50^th^ percentile) of the distribution.

Data are expressed as means ± standard deviation (SD) or numbers (percentages). Normality of the variables was tested using the Kolmogorov-Smirnov test. The data between the groups were compared with analysis of variance for continuous data or chi-square test for categorical data. Bonferroni’s post hoc tests were performed when significant differences were found to assess the magnitude of the differences.

To test the combined effect of visceral adiposity and fitness level, we tested their interactions with the interaction term for visceral adiposity*fitness level by logistic regression models for metabolic syndrome after adjusting for age and sex.

Multiple logistic regression analyses were performed to estimate the magnitude of the association of the four groups divided according to the combination of visceral adiposity and fitness level combined on the development of metabolic syndrome, after adjusting for age, sex, lifestyle factors (smoking, alcohol, and physical activity status), and BMI. The interaction between visceral adiposity and fitness level was tested at a significance level of 0.15 [25], . Hypothesis testing was two-sided at a significance level of 0.05. Statistical analyses were performed with SPSS (version 20.0; SPSS Inc., Chicago, IL, USA).

## Results

Characteristics of the participants divided into the four groups according to visceral adiposity and fitness level are shown in Table 1. The VAT/SAT ratio was approximately 0.28 in the low adiposity groups and 0.65 in the high visceral adiposity groups, and was highest in the high visceral adiposity and low fitness level group. Recovery heart rate after exercise was approximately 81.1 beats per min (bpm) in the high fitness level groups and 105.8 bpm in the low fitness level groups, and was highest in the low visceral adiposity and low fitness level group. In comparison between the groups, the high visceral adiposity groups had a tendency to be older and the low visceral adiposity groups had more males in each of their groups. Notwithstanding having greater visceral adiposity, the high visceral adiposity and high fitness level group had lower parameters of insulin resistance, insulin, and HOMA-IR than did the low visceral adiposity and low fitness level group. There were no differences in lifestyle factors (smoking, alcohol, and physical activity status) between the groups.

**Table 1:** Clinical characteristics according to visceral adiposity (VAT/SAT ratio) and fitness level (recovery heart rate).

	Low visceral adiposity	Low visceral adiposity	High visceral adiposity	High visceral adiposity	p-value^a^
	High fitness level	Low fitness level	High fitness level	Low fitness level	
	(n = 67)	(n = 58)	(n = 54)	(n = 53)	
Age (years)	31.63±8.90^‡§^	28.03±5.83^‡§^	38.06±9.52* ^†^	37.70±9.78* ^†^	<0.001
Male, n (%)	46 (68.7)^‡^	41 (70.7)^‡§^	21 (38.9)* ^†^	25 (45.3)^†^	<0.001
BMI (kg/m^2^)	28.23±3.73^†^	30.80±5.03* ^‡^	29.19±4.16^†^	29.38±4.22	0.002
Waist (cm)	94.11±9.37^†^	100.09±13.30*	98.92±11.43	98.58±11.58	0.008
Mean BP (mmHg)	89.68±10.58^†‡§^	97. 09±15.10*	99.19±9.42*	99.00±9.44*	<0.001
Visceral fat (cm^2^)	80.00±27.44^‡§^	100.05±42.81^‡§^	148.92±71.91* ^†§^	192.85±102.12* ^†‡^	<0.001
Subcutaneous fat (cm^2^)	306.42±104.94^‡^	353.94±114.96^‡§^	238.72±77.28* ^†^	286.08±108.91^†^	<0.001
VAT/SAT ratio	0.27±0.69^‡§^	0.29±0.68^‡§^	0.62±0.18* ^†^	0.67±0.21* ^†^	<0.001
Heart rate recovery	81.81±6.57^†§^	106.93±11.37* ^‡^	80.35±8.06^†§^	104.62±12.30^‡^	<0.001
Alcohol, n (%)	20 (29.9)	16 (27.6)	15 (27. 8)	18 (34.0)	0.878
Smoking, n (%)	9 (13.4)	10 (17.2)	13 (24.1)	16 (30.2)	0.227
Physical activity (MET-h/wk)	30.64±20.04	29.24±22.07	28.26±26.36	30.48±27.34	0.953
Cholesterol (mg/dl)	194.25±34.47	196.19±38.62	197.26±36.42	202.19±37.37	0.692
Triglyceride (mg/dl)	87.91±44.68^§^	122.74±67.81	127.81±49.54	165.15±183.78*	0.001
LDL (mg/dl)	121.01±32.86	123.16±36.33	123.13±35.74	121.92±34.00	0.983
HDL (mg/dl)	54.16±11.32^‡§^	50.72±11.82	48.20±10.06*	48.43±10.83*	0.010
Glucose (mg/dl)	88.76±8.11	108.43±119.52	88.76±11.23	93.36±15.36	0.247
Insulin (μU/ml)	10.02±9.27^†^	16.95±22.74* ^‡^	9.50±4.54^†^	14.35±13.23	0.012
HOMA-IR	2.24±2.15^†^	4.19±5.98* ^‡^	2.13±1.15^†^	3.03±2.61	0.005
hsCRP (mg/dL)	2.12±7.02	2.68±2.90	1.26±1.28	1.58±1.68	0.292

Abbreviations: VAT/SAT ratio, visceral adipose tissue area to subcutaneous adipose tissue area ratio; BMI, Body Mass Index; BP, Blood pressure; MET-h/wk, metabolic equivalents hour per week; LDL, low-density lipoprotein; HDL, high-density lipoprotein; HOMA-IR, Homeostasis model assessment of insulin resistance; hsCRP, high sensitive C-reactive protein.

^a^ p-values are calculated by ANOVA with Bonferroni method.

p<0.05 vs. Low visceral adiposity high fitness level; ^†^p<0.05 vs. Low visceral adiposity low fitness level; ^‡^p<0.05 vs. High visceral adiposity high fitness level; ^§^p<0.05 vs. High visceral adiposity low fitness level Variables are expressed as mean±SD for continuous variables or number (%) for categorical variables.

Visceral adiposity and fitness level were seen to have a significant interaction for the prevalence of metabolic syndrome via the interaction term between the two factors, after adjusting for age and sex (p = 0.138).

**Figure 1:**
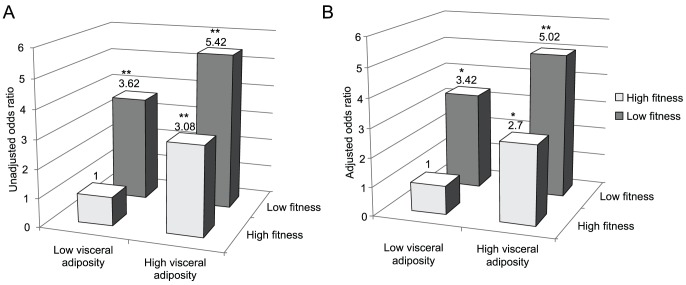
Odds ratio for metabolic syndrome according to visceral adiposity(VAT/SAT ratio) and fitness level(recovery heart rate). Adjusted odds ratio: adjusted for age, sex, smoking, alcohol, physical activity status, and body mass index. *p< 0.05, **p<0.01, calculated by multiple logistic regression analyses.

Across the four groups in regards to the association with the prevalence of metabolic syndrome, and with the most favorable group defined by low visceral adiposity and high fitness as the reference, subjects with a high visceral fat and low fitness level were associated with the highest prevalence of metabolic syndrome among all other groups (OR: 5.02, 95% CI: 1.85–13.61), after adjustment for age, sex, smoking, alcohol, physical activity status, and BMI. Furthermore, low visceral adiposity but low fitness level had a significantly higher OR for the development of metabolic syndrome than did high visceral adiposity but high fitness level, after adjustment for the covariates (OR 3.42, 95% CI: 1.27–9.19 and OR 2.70, 95% CI: 1.01–7.25, respectively) (Figure 1).

## Discussion

The objective of this study was to evaluate the joint impact of cardiorespiratory fitness and visceral adiposity on metabolic syndrome in overweight and obese adults. Our results showed that high visceral adiposity and low fitness level were cumulatively associated with metabolic syndrome. Furthermore, viscerally lean but unfit subjects were found to have a greater odds for developing metabolic syndrome than viscerally obese but highly fit individuals. These results indicate that the cardiorespiratory fitness level can alter the associations between visceral obesity and metabolic syndrome and is an important factor for reducing metabolic risk factors in overweight and obese adults.

Excessive visceral adipose tissue accumulation is one of the most important contributors to the clustering of adverse cardiometabolic profiles and metabolic syndrome that are associated with being overweight and obese [27]. Recent studies reveal that the characteristic of high visceral fat storage is a part of dysfunctional SAT expansion that leads to the functional loss of SAT as an energy deposit, resulting in ectopic fat deposition predominantly in abdominal visceral adipose tissue, liver, and skeletal muscle, and thus promoting insulin resistance [28], [29]. The concept of the VAT/SAT ratio measurement has therefore been suggested to better represent visceral fat with metabolic abnormalities, reflecting relative distribution of abdominal adipose tissue [24], [30], [31]. Indeed, the VAT/SAT ratio has been reported to have associations with metabolic syndrome, hypertension, and diabetes independent of total visceral fat volume, and with glucose intolerance, insulin resistance, and dyslipidemia [31], [32].

Cardiorespiratory fitness and metabolic syndrome may be related to the fact that unfit subjects are likely to have a high VAT [33]. Moreover, cardiorespiratory fitness is known to counteract the deleterious action of visceral adiposity [34]. In several studies, fitness level was shown to have an impact on modulating cardiovascular diseases such as diabetes as well as on mortality among overweight and obese individuals [35], [36]. While visceral fat leads to greater release of deleterious cytokines and adipokines, improving fitness leads to a greater responsiveness to advantageous adipokines and cytokines, such as leptin, adiponectin, and interleukin (IL)-10 37,38. This adaptive change in sensitivity and metabolism concurrently decreases the release of unfavorable pro-inflammatory adipokines and cytokines, such as resistin, tumor necrosis factor-α, IL-6, retinol binding protein-4, etc [39]. Additionally, the increase in fat-free lean mass and decrease in resting cortisol levels through physical exercise are demonstrated to be associated with metabolic improvements [27], [38]. As a result, fitness and fatness combine to define one’s overall health status through fitness regulating metabolic adaptations and metabolic flexibility of the viscerally obese condition. In this study, fitness was demonstrated to be a modifier of the association between visceral obesity and the prevalence of metabolic syndrome.

Reduction in insulin resistance is known to be one of the primary metabolic adaptations of fitness, which leads to improvement in insulin sensitivity and glucose tolerance for skeletal muscle and throughout the body [34], [40]. Increased expression of glucose transporter-4 protein and upregulation of glucose from enhanced skeletal muscle mitochondria enzyme activity may explain the link between cardiorespiratory fitness and improved insulin resistance [41], [42]. Our results also demonstrate that fasting serum insulin levels and the insulin resistance marker HOMA-IR were lower in the high fitness group than the low fitness group despite the high fitness group having a higher visceral adiposity.

Only a few studies have explored the combined effect of visceral adiposity and cardiorespiratory fitness on metabolic outcomes or mortality. For instance, hypertensive patients with abdominal obesity (higher waist circumference) but a high degree of fitness were shown to have lower cardiovascular disease and all-cause death than were those with normal abdominal obesity but a low level of fitness [43]. In patients with cardiovascular disease, subjects with a high waist-to-hip ratio (WHR) and a high fitness level had a lower hazards ratio for mortality than those with a low WHR and a low fitness level [44]. Our study had results in accordance with these prior studies, but was strengthened by an accurate CT measurement of visceral adiposity and by using the criteria of VAT/SAT ratio. Additionally, Rheaume et al. suggested that changes in both visceral fat accumulation and cardiorespiratory fitness were associated with changes in metabolic syndrome components and that the two factors are both important for the maintenance of healthy cardiometabolic risk profiles, which is consistent with the results from our study [45]. Here, we compared these associations for individuals by stratifying their central adiposity by fitness level, permitting more rigorous analyses of combined subgroups than adjustment for either variable alone [43].

There were some limitations to our study. The directionality and causality of the results of cross-sectional analyses could not be determined with certainty. The 3-minute step test was used to measure fitness level instead of a direct measure of maximal oxygen uptake (VO_2_ max). However, the correlation between the 3-minute step test results and VO_2_ max has been validated in many studies so far, and the 3 minute step test is known to be a relatively quick and easy method used in clinical settings and in epidemiologic studies since the early 20^th^ century [46], [47]. Also, the HOMA-IR index was used instead of direct measurement for the representation of insulin resistance. Nonetheless, HOMA-IR has been validated as a reliable and clinically useful marker for assessing beta-cell function and insulin resistance [48]. Finally, as the sample was composed of more young to middle-aged adults, it may be difficult to generalize the data and results to older populations with a greater tendency toward higher visceral adiposity accumulation. Further studies are warranted to clearly identify the combined effect of fitness and visceral fatness to metabolic characteristics in an older and larger population.

## Conclusions

In conclusion, visceral obesity and fitness level are cumulatively associated with the prevalence of metabolic syndrome in healthy overweight and obese adults. This suggests that cardiorespiratory fitness is a significant modifier of the relation between visceral adiposity and adverse metabolic outcomes, and the effects of improvement in fitness can be an important target for maintenance of the overall healthy metabolic characteristics in overweight and obese individuals.
